# Chemotherapy alone is an alternative treatment in treating localized primary ocular adnexal lymphomas

**DOI:** 10.18632/oncotarget.18500

**Published:** 2017-06-15

**Authors:** Wei-Li Ma, Ming Yao, Shu-Lang Liao, Jih-Luh Tang, Yao-Ching Wang, Sung-Hsin Kuo, Ann-Lii Cheng

**Affiliations:** ^1^ Department of Oncology, National Taiwan University Hospital, Yun-Lin Branch, Yun-Lin, Taiwan; ^2^ Department of Oncology, National Taiwan University Hospital and National Taiwan University College of Medicine, Taipei, Taiwan; ^3^ Division of Hematology, Department of Internal Medicine, National Taiwan University Hospital, Taipei, Taiwan; ^4^ Department of Ophthalmology, National Taiwan University Hospital, Taipei, Taiwan; ^5^ National Taiwan University Cancer Center, National Taiwan University College of Medicine, Taipei, Taiwan; ^6^ Graduate Institute of Oncology, National Taiwan University College of Medicine, Taipei, Taiwan; ^7^ Cancer Research Center, National Taiwan University College of Medicine, Taipei, Taiwan; ^8^ Department of Biomedical Imaging and Radiological Science, College of Health Care, China Medical University, Taichung, Taiwan

**Keywords:** ocular adnexal lymphomas, chemotherapy, radiotherapy, event-free survival, overall survival

## Abstract

This study investigated the treatment efficacy and long-term adverse effects of various treatment modalities for primary ocular adnexal lymphomas (POALs). We retrospectively reviewed 107 patients who received first-line chemotherapy, radiotherapy, and other treatment modalities from 1990 to 2015. Nighty-three (87%) patients were diagnosed with mucosa-associated lymphoid tissue (MALT) lymphoma, with the orbit being the most common site (49 patients, 46%). Among 91 patients with stage I–IIE1 disease, 26 underwent chemotherapy, 34 underwent radiotherapy, and 31 received other treatment modalities. For chemotherapy, radiotherapy, and other treatment modalities, the 5-year event-free survival (EFS) rates were 90.0%, 89.7%, and 85.8% and the 5-year overall survival (OS) rates were 100%, 90.4%, and 87.5%, respectively. Moreover, among 80 patients with stage I–IIE1 MALT lymphoma, the complete remission, 5-year EFS and OS rates were not significantly different between patients receiving chemotherapy and those receiving radiotherapy. Among 16 patients with stage IIE2–IVE disease, the 5-year EFS rates for chemotherapy alone (*n* = 11) and combined radiotherapy and chemotherapy (*n* = 5) were 61.7% and 80%, respectively, whereas the 5-year OS rate for both groups was 80.0%. Neutropenia (15.2%) was the most common side effect in patients who received chemotherapy, whereas cataract (16.3%) was the most common late sequela in patients who received radiotherapy. Multivariate analysis revealed that old age (> 60 y) and an advanced stage (stage III/IV) were prognostic factors for poor OS. Our results indicate that chemotherapy yields satisfactory disease control and fewer side effects, and acts as an alternative therapy for patients with localized POALs.

## INTRODUCTION

Primary ocular adnexal lymphomas (POALs) present in the orbit, extraocular muscle, eyelids, lacrimal gland, or conjunctiva and represent up to 1% of all non-Hodgkin lymphomas [1, 2]. Mucosa-associated lymphoid tissue (MALT) lymphoma is the most common type. In addition, diffuse large B-cell lymphoma (DLBCL) with or without histological evidence of MALT origin or follicular lymphoma (FL) has been reported [3, 4]. Treatment strategies for localized POALs remain controversial. Although a proportion of patients with a histologic diagnosis of low-grade MALT lymphoma of POAL (MALT-POAL) can be treated using doxycycline (conventionally used for eradicating *Chlamydia psittaci* [5, 6]), local radiotherapy is the standard first-line treatment for patients with MALT-POAL in Western countries [7]. Previous studies have demonstrated that radiotherapy provides an immediate effect and excellent local control of indolent POALs, including MALT lymphoma and FL; however, late recurrences have been reported [8–10]. Notably, local radiotherapy to the ocular adnexal areas possibly causes acute and late side effects, such as cataract, dry eyes, keratitis, and retinopathy [11, 12].

In contrast to local radiotherapy, systemic chemotherapy with a single alkylating agent (chlorambucil) or a combination of cyclophosphamide, vincristine, and prednisolone (CVP) has been administered to treat localized (stage IE to IIE1) MALT-POAL, with a reported complete remission (CR) rate of approximately 75% [13, 14]. In addition to systemic chemotherapy, studies have reported that rituximab monotherapy serves as the first-line or second-line therapy for low-grade POALs (included MALT lymphoma and FL), providing a CR rate of approximately 30%–50% and disease-free status [15–18]. However, the long-term clinical outcomes of combined chemotherapy and rituximab in patients with localized POALs have rarely been described.

In this study, we aimed to identify the clinical outcomes of frontline systemic chemotherapy with or without rituximab in patients with all POAL stages. In addition, we compared the treatment efficacy and long-term adverse effect of systemic chemotherapy (with and without rituximab) with those of radiotherapy alone in patients with localized POALs.

## RESULTS

### Patient characteristics and treatment modalities

In total, 58 men and 49 women with a median age of 58 years were included. Among them, 93 patients (87%) had MALT lymphoma, 5 (5%) had high-grade transformed MALT lymphoma (renamed DLBCL with MALT lymphoma, DLBCL (MALT)), and 9 (8%) had DLBCL. The orbit was the most commonly involved site (49 patients, 46%), followed by the conjunctiva (42 patients, 39%) and lacrimal gland (16 patients, 15%). Nighty-one patients had stage IE–IIE1 and 16 had stage IIE2–IVE disease (Table 1). The median age and distributions of sex, orbital laterality, primary lymphoma location, and lymphoma type did not differ between these 2 groups (stages IE–IIE1 vs. stages IIE2–IVE) (Table 1).

**Table 1:** Comparison of the clinical characteristics of patients with stage IE–IIE1 or IIE2–IV POALs

	Stage I–IIE1	Stage IIE2–IV	*P*
**Number of patients**	91	16	
**Age at diagnosis**			0.248
Median	57	65.5	
Range	22–102	29–84	
**Sex**			0.872
Male	48	10	
Female	43	6	
**Orbital laterality**			0.904
Right	41	8	
Left	34	4	
Bilateral	16	4	
**Location**			0.980
Orbital	40	9	
Conjunctiva	39	3	
Lacrimal gland	12	4	
**Pathology**			0.468
MALT lymphoma	80	13	
DLBCL(MALT)	4	1	
DLBCL	7	2	

Abbreviations: POALs, primary ocular adnexal lymphomas; MALT, mucosa-associated lymphoid tissue; DLBCL, diffuse large B-cell lymphoma; DLBCL(MALT), DLBCL with histologic evidence of MALT lymphoma by WHO classification.

The treatment regimens are detailed in Table 2. Among the patients with stage IE–IIE1 disease, 26 received chemotherapy (20 MALT lymphoma, 1 DLBCL (MALT), and 5 DLBCL); 34 received radiotherapy (29 MALT lymphoma, 3 DLBCL (MALT), and 2 DLBCL); and 31 received other treatment modalities. The radiation prescription dose ranged from 30 to 50 Gy (median dose: 40 Gy) in daily fractions of 1.8–2.0 Gy. Chemotherapy or rituximab courses ranged from 2 to 8 cycles, and prophylactic granulocyte colony stimulating factor (G-CSF) was used on the basis of attending physicians’ judgment. Among the patients who received other treatment modalities, 26 underwent surgery alone followed by observation, 4 received combined radiotherapy and chemotherapy, and 1 received intralesional rituximab injection. Among the patients with stage IIE2–IVE disease, 11 received chemotherapy and 5 received combined radiotherapy and chemotherapy.

**Table 2:** Treatment modalities of patients with stage IE–IIE1 and stage IIE2–IV POALs

	Number of patients
**Stage IE–IIE1**	
**Chemotherapy**	26
Low-dose alkylating chemotherapy with/without rituximab	8
CHOP-based chemotherapy	5
R-CHOP–based chemotherapy	13
**Radiotherapy**	34
2D technique	30
3D conformational technique	2
Intensity-modulated radiotherapy	1
Volumetric modulated arc therapy	1
**Other treatments**	31
Surgery alone	26
Radiotherapy + low-dose alkylating chemotherapy with/without rituximab	1
Radiotherapy + CHOP-based chemotherapy	2
Radiotherapy + R-CHOP–based chemotherapy	1
Intralesional rituximab injection	1
**Stage IIE2–IV**	
**Chemotherapy**	11
Low-dose alkylating chemotherapy with/without rituximab	5
CHOP-based chemotherapy	2
R-CHOP-based chemotherapy	4
**Combined chemotherapy and radiotherapy**	5
Radiotherapy + low-dose alkylating chemotherapy with/without rituximab	3
Radiotherapy+ CHOP-based chemotherapy	2

Abbreviations: POALs, primary ocular adnexal lymphomas; CHOP, cyclophosphamide, doxorubicin, vincristine, and prednisolone; R-CHOP, rituximab + CHOP.

### Treatment response and clinical outcome of patients with stage IE to IIE1 POALs

**Figure 1:**
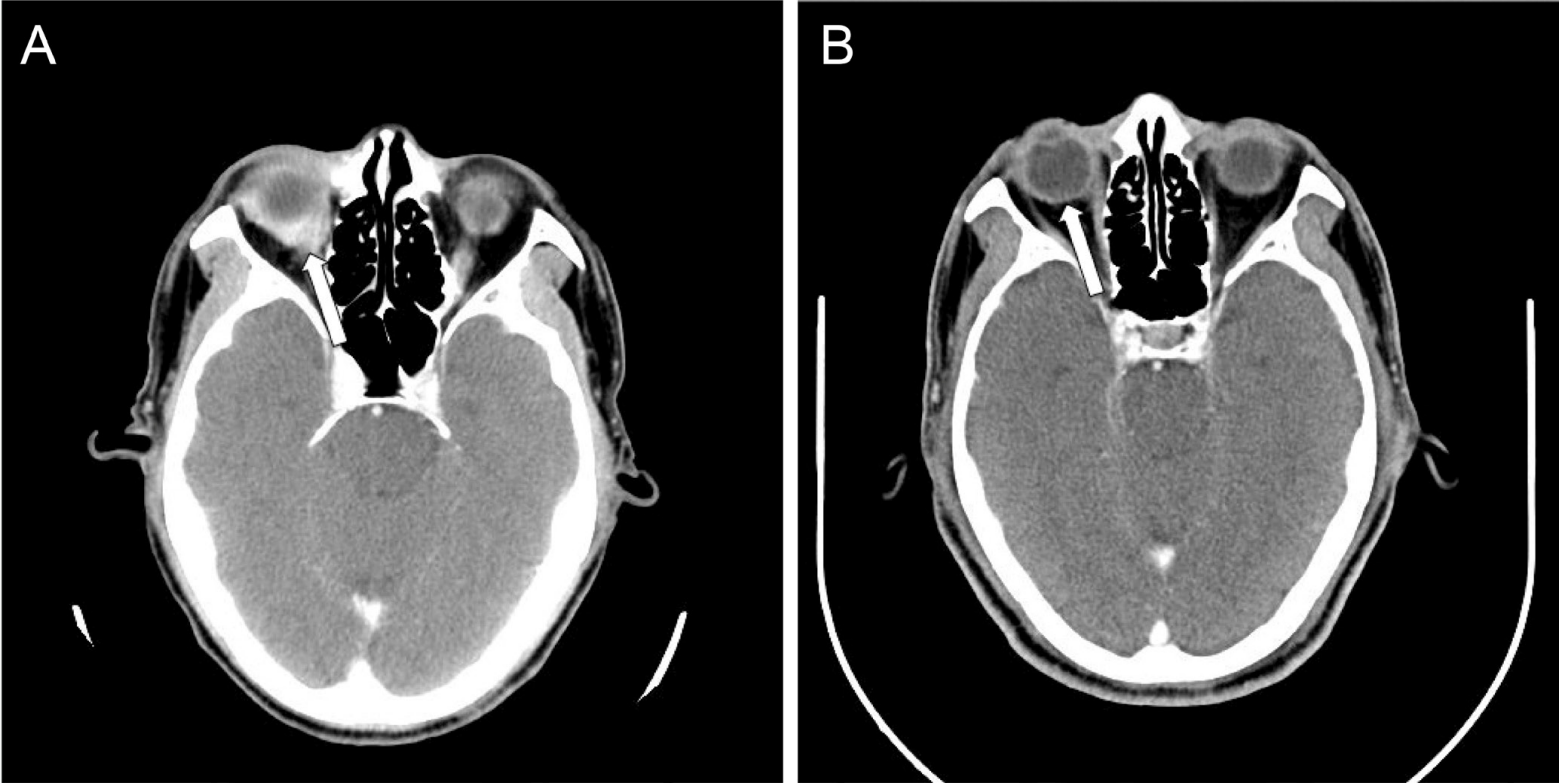
The CT scan of the brain with contrast showing (**A**) before treatment, the enhanced soft tissue infiltrates the right eyeball (arrow); and (**B**) after four cycles of R-CHOP, the tissue resolved with CR.

**Figure 2:**
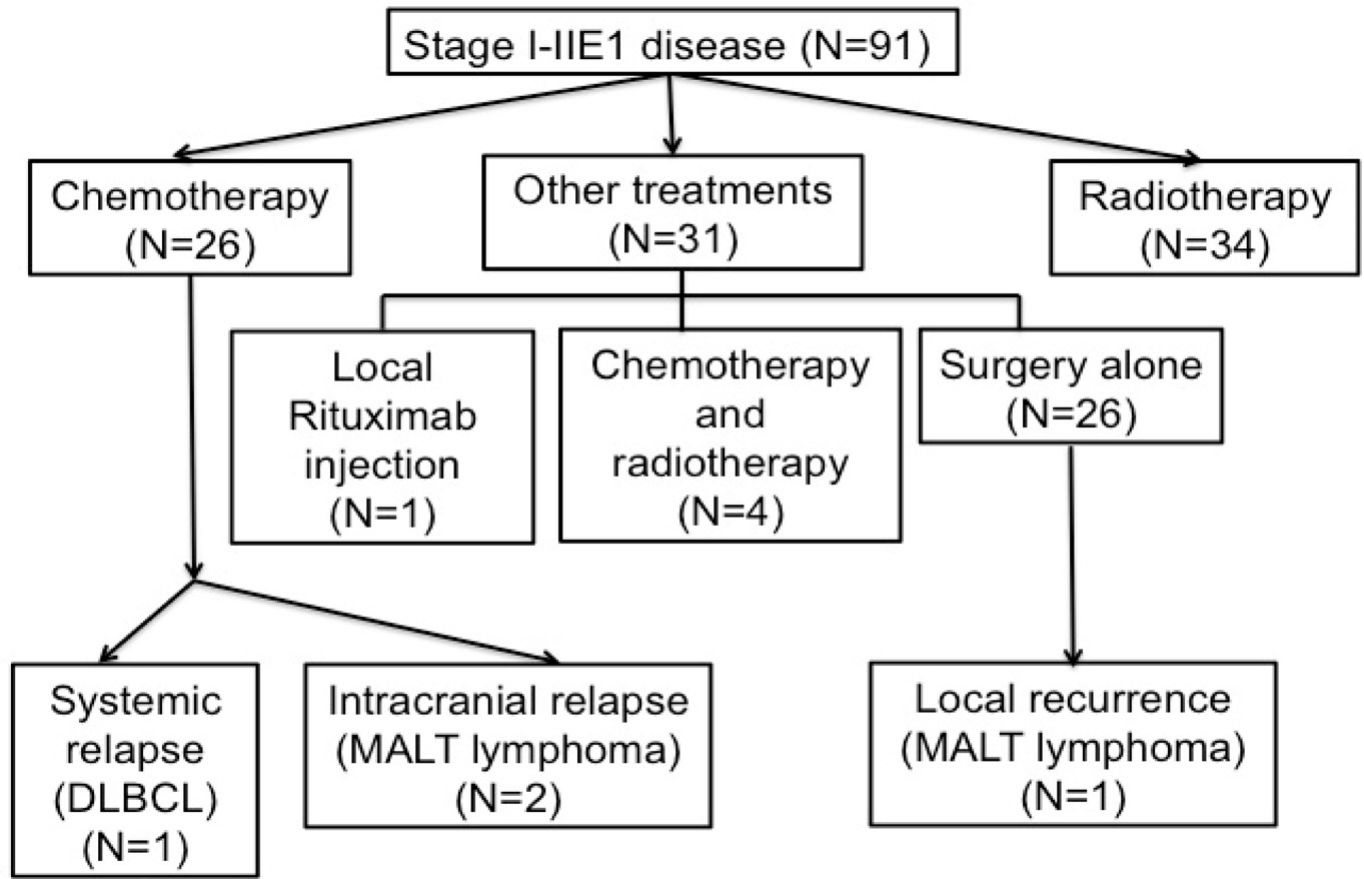
Flow diagram of patients with stage I–IIE1 primary ocular adnexal lymphoma The patients are listed from intial treatment to disease relapse.

Among the patients with stage IE–IIE1 POALs, the CR rates of those receiving chemotherapy, radiotherapy, and other treatment modalities were 87.5%, 91.2%, and 67.8%, respectively. Among the patients receiving chemotherapy, the CR rates of those receiving oral alkylating agents with or without rituximab, the cyclophosphamide, doxorubicin, vincristine, and prednisolone (CHOP)-based regimen, and the rituximab (R)-CHOP-based regimen were 87.5%, 60.0%, and 76.9%, respectively (Figure 1) (Supplementary Table 1). During a median follow-up period of 54.7 months, 4 (4%) of the 91 patients with stage I–IIE1 POALs experienced a relapse (Figure 2). One patient with conjunctival MALT lymphoma underwent first-line surgical excision. The patient had a local recurrence and underwent re-excision. Another patient with orbital DLBCL received first-line R-CHOP chemotherapy and had a retroperitoneal relapse. The patient then received a second-line regimen of rituximab, etoposide, methylprednisolone, high-dose cytarabine, and cisplatin and experienced CR. The other 2 patients had orbital MALT lymphoma; one received daily low-dose cyclophosphamide with rituximab, whereas the other received R-CHOP chemotherapy. They experienced a relapse in the central nervous system and received high-dose methotrexate treatment to achieve CR in the brain.

**Figure 3:**
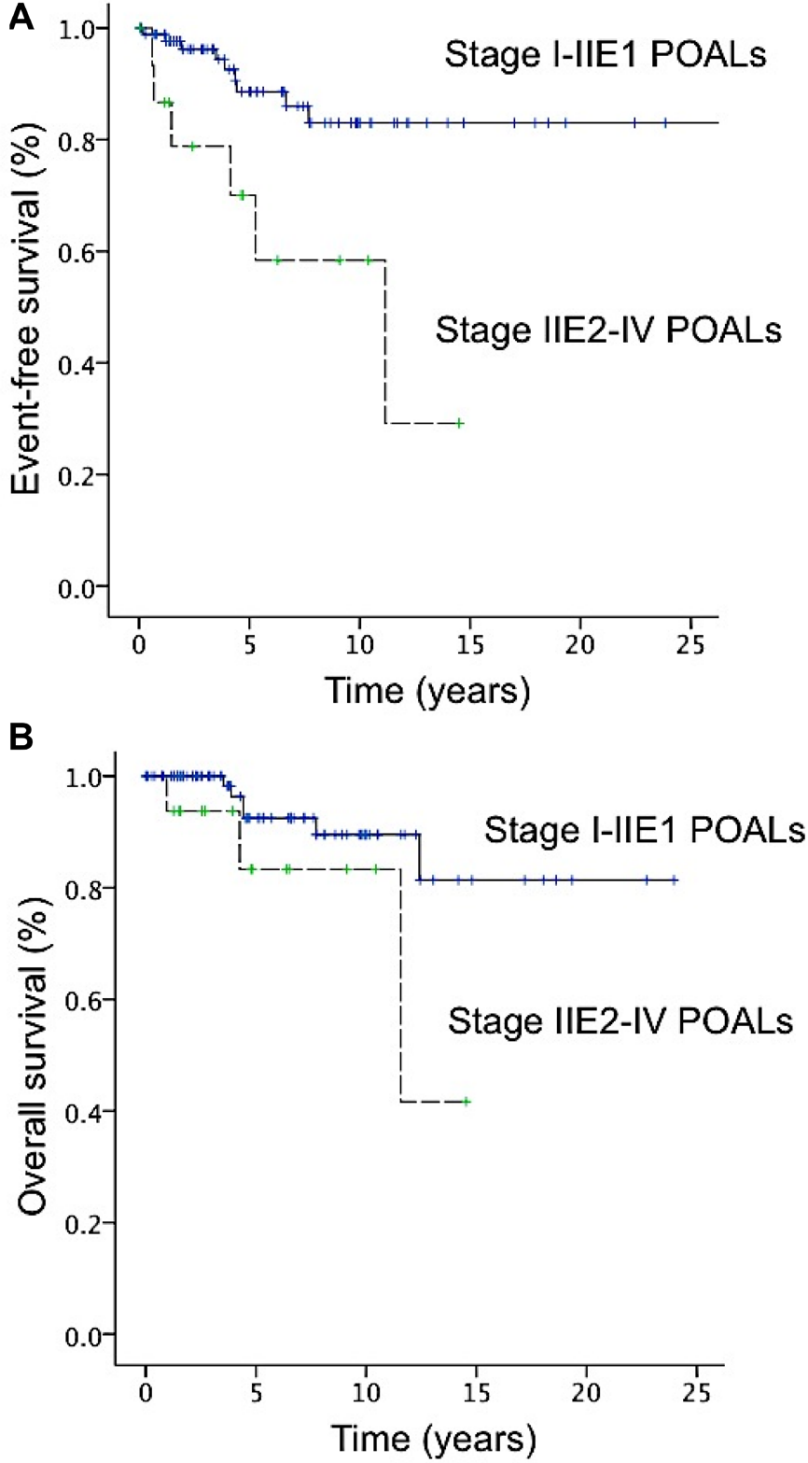
Curves of event-free surival (EFS) and overall survival (OS) of patients with primary ocular adnexal lymphoma (**A**) The 5-year EFS rates for stage I–IIE1 and IIE2–IV disease were 88.6% and 70.0%, respectively. (**B**) The 5-year OS rates for stage I–IIE1 and IIE2–IV disease were 92.5% and 83.3%, respectively.

The 5-year event-free survival (EFS) and overall survival (OS) rates of the patients with stage IE–IIE1 POALs were 88.6% and 92.5%, respectively (Figure 3). Among patients with localized (stage I-IIE1) POALs, we found that the 5-year EFS rates of patients with MALT lymphoma, DLBCL (MALT), and DLBCL were 92.7%, 50.0%, and 80.0%, respectively; the 5-year OS rates of the patients with MALT lymphoma, DLBCL (MALT), and DLBCL were 95.4%, 50.0%, and 100%, respectively. Because of the small number of patients with DLBCL (MALT), we divided localized patients into two subgroups, MALT lymphoma and DLBCL (also including DLBCL (MALT)). We found that patients with MALT lymphoma had a better 5-year EFS than those with DLBCL (including DLBCL (MALT); 92.7 vs. 64.8, *P* = 0.05). However, the OS was not different between these two subgroups (MALT lymphoma vs. DLBCL; 95.4% vs. 77.8%, *P* = 0.176).

**Figure 4:**
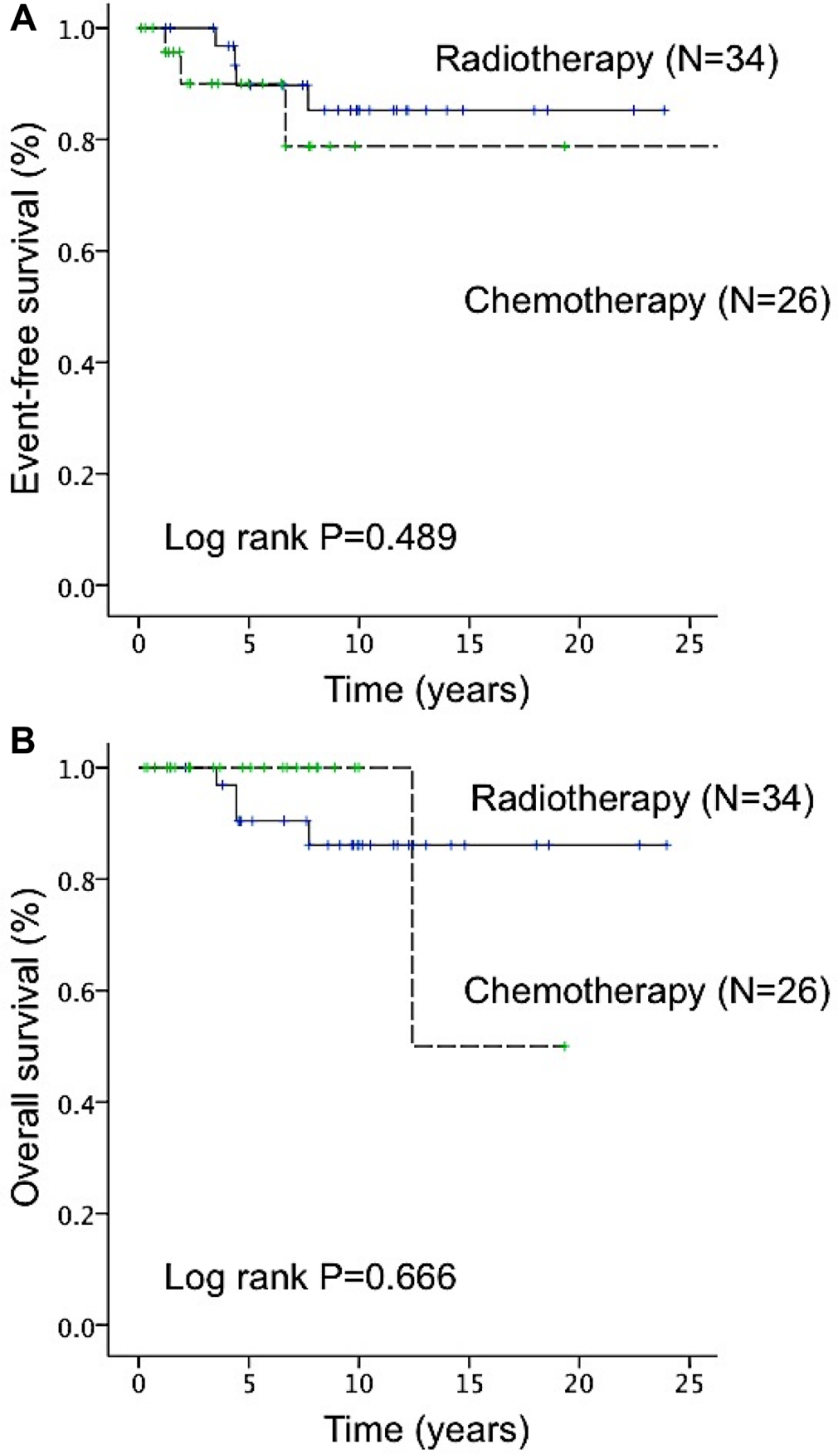
Difference in event-free survival (EFS) and overall survival (OS) between radiotherapy and chemotherapy (included rituximab-based immunochemotherapy) in patients with stage IE–IIE1 primary ocular adnexal lymphoma (**A**) The 5-year EFS rate did not significantly differ between patients who underwent radiotherapy and those who underwent chemotherapy or rituximab-based immunochemotherapy (89.7% vs 90.0%, *P* = 0.489). (**B**) The 5-year OS rate did not significantly differ between patients who underwent radiotherapy and those who underwent chemotherapy or rituximab-based immunochemotherapy (90.4% vs 100.0%, *P* = 0.666).

Furthermore, among patients with stage IE-IIE1 POALs, the 5-year EFS rates of the patients with chemotherapy or rituximab-based immunochemotherapy, radiotherapy, and other treatment modalities were 90.0%, 89.7%, and 85.8%, respectively; the 5-year OS rates of the patients with chemotherapy or rituximab-based immunochemotherapy, radiotherapy, and other treatment modalities were 100%, 90.4%, and 87.5%, respectively. The 5-year EFS and OS rates of the patients undergoing different chemotherapy regimens or rituximab-based immunochemotherapy are listed in Supplementary Table 1. Among patients with stage IE-IIE1 POALs, patients receiving systemic chemotherapy or rituximab-based immunochemotherapy had similar 5-year EFS (*P* = 0.489) and OS rates (*P* = 0.666) when compared with those receiving radiotherapy (Figure 4). Notably, patients with localized POALs who received an oral alkylating agent alone had a CR rate of 80%, a 3-year EFS of 100%, and a 3-year OS of 100%.

### Treatment response and clinical outcome of patients with stage IE to IIE1 MALT lymphoma of POALs

Because DLBCL is traditionally treated with chemotherapy or rituximab-based immunochemotherapy, we analyzed whether the efficacy of first-line systemic chemotherapy was the same as radiotherapy for localized MALT lymphoma when patients do not qualify for radiotherapy or when patients were afraid of potential long-term side effects of radiotherapy. The detailed information of the first-line treatment, including radiotherapy, chemotherapy or rituximab-based regimens of 80 patients with stage IE to IIE1 MALT lymphoma of POALs are listed in Supplementary Table 2. We found that the CR rate for patients with radiotherapy, chemotherapy, and rituximab-based chemotherapy were 90%, 62.5%, 83.3%, respectively.

As shown in Supplementary Figure 1, we found that the 5-year EFS rate was not significantly different between patients receiving chemotherapy or rituximab-based immunochemotherapy and those receiving radiotherapy (94.4% vs. 96.0%, *P* = 0.250). In addition, the 5-year OS rate for patients receiving chemotherapy or rituximab-based immunochemotherapy and those receiving radiotherapy was 100% and 96.3%, respectively (*P* = 0.511) (Supplementary Figure 1).

Nevertheless, among 26 patients with MALT lymphoma of POAL who underwent surgery alone, one developed a local recurrence after a follow-up period of 2.3 months; however, the remaining patients remained disease free after a follow-up period of up to 10 years.

### Treatment response and clinical outcome of patients with stage IIE2 to IV POALs

The 5-year EFS and OS rates of the patients with stage IIE2–IV disease were 70.0% and 83.3%, respectively. No significant differences were observed in the 5-year EFS (61.7% vs 80.0%, *P* = 0.388) or OS rates (80.0% vs 80.0%, *P* = 0.721) between patients receiving systemic chemotherapy or rituximab-based immunochemotherapy alone and those receiving systemic chemotherapy combined with local radiotherapy. The CR rate and 5-year EFS and OS rates of patients (all stages) receiving chemotherapy are listed according to the regimen in Supplementary Table 1.

### Prognostic factors

In univariate analysis, an age > 60 years (*P* = 0.025), high-grade lymphoma components (*P* = 0.23), increased serum LDH (*P* = 0.011), Ann Arbor stages III–IV (*P* = 0.008), and advanced American Joint Committee on Cancer (AJCC) TN stages (T3: preseptal eyelid involvement, T4: extending beyond the orbit to adjacent structures, (*P* = 0.006); N: lymph node involvement (*P* = 0.006) were significantly associated with shorter EFS (Table 3). The primary lymphoma site (conjunctiva, orbital, or lacrimal gland), and advanced AJCC M stage were not associated with poor EFS (Table 3). An age > 60 years was a characteristic finding for predicting poor EFS in multivariate analysis (Table 3). In addition, an age > 60 years and Ann Arbor stages III–IV were significantly associated with poor OS in univariate and multivariate analyses (Table 4).

**Table 3:** Univariate and multivariate analyses of event-free survival after frontline treatments in all patients with POALs

Factors	Relative risk	95% Cl	*P* value
**Univariate analysis**			
Age > 60 years	3.720	1.184–11.691	**0.025**
Locations			
Conjunctiva	1		
Orbital	0.311	0.062–1.559	0.155
Lacrimal gland	1.016	0.273–3.788	0.981
High-grade components	3.483	1.185–10.237	**0.023**
LDH elevation	3.281	1.357–10.759	**0.011**
Ann Arbor stage III–IV disease	4.293	1.464–12.590	**0.008**
AJCC stage T3 and T4 disease	4.254	1.503–12.042	**0.006**
AJCC stage with N1–4 disease	5.191	1.608–16.764	**0.006**
AJCC stage with M1 disease	2.538	0.715–9.012	0.150
**Multivariate analysis**			
Age > 60 years	4.441	1.133–17.400	**0.032**

Abbreviations: POALs, primary ocular adnexal lymphomas; CI, confidence interval; LDH, lactate dehydrogenase; AJCC, American Joint Committee on Cancer.

**Table 4:** Univariate and multivariate analyses of overall survival in all patients with POALs

Factors	Relative risk	95% Cl	*P* value
**Univariate analysis**			
Age > 60 years	9.923	1.240–79.387	**0.031**
Locations			
Conjunctiva	1		
Orbital	0.140	0.012–1.604	0.114
Lacrimal gland	0.901	0.174–4.677	0.901
High-grade components	3.021	0.748–12.200	0.121
LDH elevation	2.672	0.661–10.799	0.168
Ann Arbor stage III–IV disease	4.039	1.005–16.226	**0.049**
AJCC stage T3 and T4 disease	3.169	0.779–12.895	0.107
AJCC stage with N1–4 disease	2.061	0.245–17.345	0.506
AJCC stage with M1 disease	2.733	0.566–13.191	0.211
**Multivariate analysis**			
Age > 60 years	37.275	2.364–587.675	**0.010**
Ann Arbor stage III–IV disease	35.884	1.162–1108.528	**0.041**

Abbreviations: POALs, primary ocular adnexal lymphomas; CI, confidence interval; LDH, lactate dehydrogenase; AJCC, American Joint Committee on Cancer.

### Treatment-related side effects

We analyzed acute and late toxicities in the chemotherapy (included rituximab-based immunochemotherapy) and radiotherapy groups. Among the 46 patients with chemotherapy, 17 received oral alkylating chemotherapy with or without rituximab and 29 received CHOP or R-CHOP-based regimens. No chemotherapy-related side effects were observed in the oral alkylating chemotherapy group. In the CHOP or R-CHOP chemotherapy group, 7 patients (15.2%) had neutropenia, 2 (4%) had anemia, and 6 (13%) had increased liver enzymes. These acute side effects were manageable and reversible, and no secondary hematologic malignancy was reported during a follow-up period of 22.6 years.

Among the 43 patients who received local radiotherapy, 11 had late ophthalmologic complications: 3 (7%) had keratitis, 5 (12%) had cataract, and 1 (2%) had retinopathy. One patient had both keratitis and cataract, whereas another patient had both cataract and retinopathy. In addition, cataract was the most common late ophthalmological complication, occurring in 7 patients. Some patients received a total radiation dose of 50 Gy, and lens shielding was not provided in early years, leading to retinopathy in 2 patients.

## DISCUSSION

In this study, we observed that cytotoxic chemotherapy and chemotherapy combined with rituximab were effective in treating localized and disseminated POALs, most of which received a histological classification of MALT lymphoma. In this study, we reported that among patients with localized POALs (stage I-IIE1), low-dose oral alkylating agent alone had a CR rate of 80%, a 3-year EFS of 100%, and a 3-year OS of 100%. Considering that low-dose alkylating chemotherapy is less toxic than a combined chemotherapy regimen, such as CVP or CHOP, our results indicate that in addition to radiotherapy, low-dose alkylating chemotherapy alone can be an alternative first-line treatment for patients who are concerned about potential late ophthalmic complications of radiotherapy, or whose conditions are not suitable for radiotherapy.

Our results revealed that radiotherapy alone with a median dose of 40 Gy provided 5-year EFS and OS rates of 89.7% and 90.4%, respectively, for patients with stage IE–IIE1 POALs. These findings are consistent with those of previous studies that reported that local radiotherapy of at least 30 Gy resulted in effective local control with 5-year progression-free survival (PFS) and OS rates of 65.5%–93.0% and 73.6%–100%, respectively [8–10, 12, 19, 20]. Some studies have reported an association of the histological evidence of MALT lymphoma with improved PFS and OS rates [19, 20]. However, the major late ophthalmological complications occurred with a radiotherapy dose of more than 30–35 Gy [21]. Our results and those of other studies have revealed that cataract, dry eye, and keratitis are the most common late radiotherapy-related adverse ophthalmological effects [11, 12]. A decreased radiotherapy dose of 24–25 Gy and deployment of a lens shield can reduce late sequelae without compromising POALs treatment efficacy [11, 22]. Fasola et al. reported that among 20 patients with POALs (95% were of the FL or MALT subtype), the administered low-dose radiotherapy (2 fractions of 2.0 Gy) resulted in an overall response rate of 96% and 2-year local PFS of 100%, without late ophthalmological complications [23]. In addition, the same low-dose radiotherapy (2 fractions of 2.0 Gy) provided a 2year local PFS of 100% and a distant PFS of 68.6 %, but without the late complication of cataract in 7 patients with 8 lesions of POALs [24]. These findings indicate that low-dose radiotherapy can provide effective local control, with few late ophthalmological complications, whereas distant relapse should be considered when using low-dose radiotherapy for POALs.

Studies have examined the efficacy of different chemotherapy regimens in treating patients with localized POALs [13, 14, 25, 26]. For example, the administration of a single alkylating agent (chlorambucil) produced a CR rate of 79% [13]. Moreover, combined CVP regimens provided a CR rate of 76.2% and a 5-year PFS rate of 66% [14]. Rituximab alone exhibited moderate activity in treating POALs, particularly MALT lymphoma, with a CR rate of approximately 30%–50% and a median time to progression of 5 months [15–18]. In addition, some studies reported that prolonged administration of rituximab to 8 cycles or administration of rituximab for maintenance can increase the treatment response rate [27, 28]. Rigacci et al reported that when rituximab was combined with chlorambucil, the CR rate increased to 89% in low-grade POALs [29]. Compared to local radiotherapy for localized POALs, Mino et al and Paik et al also reported that chemotherapy, rituximab alone, or immunochemotherapy is an effective treatment for MALT-POAL [28, 30]. We observed a CR rate of 76.9% and a 5-year OS rate of 66.7% in 13 patients with stage I–IV POAL after the administration of an oral alkylating agent with or without rituximab; these rates are consistent with the results of Rigacci et al [29].

In the present study, we analyzed the CR rate and the clinical outcomes of a total of 37 patients who received chemotherapy or rituximab-based immunochemotherapy. Among patients with localized POALs, we found that the CR rate and the 5-year EFS and OS were not different between patients receiving chemotherapy or rituximab-based immunochemotherapy (rituximab alone or rituximab plus chemotherapy) and those receiving radiotherapy. Although the number of enrolled patients was small, most of our patients received oral alkylating agents (chlorambucil) or rituximab-based immunochemotherapy and had long-term follow-up. Because most patients with localized POALs are treated with radiotherapy, there were very few patients receiving systemic chemotherapy as first-line treatment. However, for patients who cannot receive radiotherapy or did not respond to radiotherapy, an alternative treatment with similar efficacy and fewer side effects, such as chemotherapy or rituximab-based immunochemotherapy is necessary. Combining our results and that of 10 other investigators (Table 5), a CR was achieved in 80% of patients with stage IE-IIE1 MALT lymphoma who received chlorambucil and in 83.3% of patients who received rituximab-based regimens. Kiesewetter et al. also reported that among 16 patients with stage IE-IIE1 POALs, immunochemotherapy regimens (included lenalidomide, bortezomib, oxaliplatin, CHOP, rituximab, or rituximab-based regimens) resulted in 81.3% overall response rate (9 cases, CR; 4 cases, partial remission [PR]) [31]. These findings indicate that in addition to radiotherapy, chemotherapy or rituximab-based regimen is an effective treatment strategy for patients with localized POALs, including those with MALT lymphoma histology.

**Table 5:** Summary of treatment outcomes after chemotherapy in patients with POALs

Studies	Patient no.	Stage	Histology types	Regimens	Treatment response	Survival
Ben Simon, et al. [13]	33	IE	MALT lymphoma	Chlorambucil	26 (79%):CR 7 (21%):PR	
Song, et al. [14]	21	I-IIE	MALT lymphoma	CVP	16 (76%):CR 5 (24%):PR	5-year PFS: 66%
Nückel, et al. [15]	2	IE	MALT lymphoma	Rituximab	1 (50%):CR 1 (50%):PR	
Ferreri, et al. [16]	8	IE: 4, IV: 4	MALT lymphoma	Rituximab	3 (37.5%):CR 2 (25%):PR	
Tuncer, et al. [17]	10	IE	MALT lymphoma: 9; FL: 1	Rituximab	5 (50%): CR 5 (50%): PR	
Sokol, et al. [18]	2	IE	MALT lymphoma	Rituximab	1 (50%):CR 1 (50%):PR	5-year PFS: 100%
Annibal, et al. [27]	6	IE	MALT lymphoma	Rituximab	4 (67%):CR 2(33%):PR	
Mino, et al. [28]	10	I-IIE	MALT lymphoma	Rituximab	10 (100%):CR	
Rigacc, et al. [29]	9	IE: 8, IV: 1	MALT lymphoma: 8; FL:1	Rituximab+ chlorambucil	8 (89%):CR 1 (11%):PR	
Paik, et al. [30]	9	I-IIE: 7 IVE: 2	MALT lymphoma	CVP, CHOP, R-CVP, R-CHOP	9 (100%):CR	
Present study	20	I-IIE1	MALT lymphoma	Total: 20 Chlorambucil: 5 Rituximab-based: 12 CHOP: 3	15 (75%):CR 4 (80%):CR 10 (83%):CR 1 (33.3):CR	5-year EFS: 94.4% 5-year OS: 100%

Abbreviations: POALs, primary ocular adnexal lymphomas; MALT, mucosa-associated lymphoid tissue; FL, follicular lymphoma; DLBCL (MALT): diffuse large B-cell lymphoma with histologic evidence of MALT lymphoma; CVP, cyclophosphamide, vincristine, and prednisolone; CHOP, cyclophosphamide, vincristine, doxorubicin, prednisolone; R-CVP, rituximab, cyclophosphamide, vincristine, and prednisolone; R-CHOP, rituximab, cyclophosphamide, vincristine, doxorubicin, prednisolone; CR, complete remission; PR, partial remission; PFS, progression-free survival.

Although the fluorine 18 fluoro-deoxyglucose positron emission tomography (FDG-PET) provides more precise staging for lymphoma, computed tomography (CT) or magnetic resonance imaging (MRI) provided higher sensitivity than FDG-PET for local ocular adnexal lesions of POALs. For example, English et al. demonstrated that FDG-PET detected systemic diseases more reliably than CT scan alone, whereas FDG-PET was less sensitive than CT scan for detecting localized POALs [32]. In addition, Zanni et al. revealed that among patients with POALs, especially those with non-conjunctival sites of lymphoma, the sensitivity of FDG-PET was lower than that of MRI scan in the assessment of POALs [33]. Considering that 85% of our patients had localized disease (stage I–IIE1), whole-body CT scan, including orbital MRI, at initial staging is adequate, although only 5 of our 91 localized POAL patients underwent FDG-PET. However, we cannot exclude the possibility that a proportion of our patients with localized POALs (stage IE-IIE1) may be re-staged to stage IIE2-IV diseases if FDG-PET was incorporated as an initial staging method.

Zucca et al investigated prognostic factors for patients with nongastric MALT lymphoma in a retrospective study. They reported that Ann Arbor stage IV was a prognostic factor for poor OS, whereas classifying patients into groups of intermediate to high risk on the basis of international prognostic index scores had a negative influence on PFS in multivariate analysis [34]. Martinet et al revealed that among patients with POALs who received radiotherapy, a young age and low-grade lymphoma were associated with improved OS and disease-free survival in multivariate analysis [35]. In the multivariate analyses, we found that an age > 60 years was an independently poor prognostic factor for EFS, whereas elevation of LDH, and stages III-IV lost significances in EFS. In addition, an age > 60 years and Ann Arbor stages III–IV were significantly associated with poor OS. Our findings are lined with a retrospective analyses of 182 patients with MALT lymphoma of POALs (80% of them were stage I) reporting that an age > 60 years was independently associated with the shorter progression-free survival [36]. Meunier et al. also showed that age greater than 59 years was significantly associated with poor OS for ophthalmologic and intraocular NHL populations in the multivariate analysis [37]. However, the precise mechanism responsible for the poorer prognosis in older patients with POALs remains to be clarified. Studies using the TNM system to evaluate the prognosis of POALs reported an association of T3, T4, N, and M stages with poor outcomes [38–40]. However, an advanced TNM stage of POALs did not affect the prognosis in our cohort.

Our study had some limitations. First, it was a retrospective study, and some patients did not strictly follow the treatment protocols. Second, the study was conducted between 1990 and 2015, and improved radiation techniques may affect outcomes and reduce side effects. However, we provided long-term favorable outcomes by using systemic chemotherapy alone to treat localized or disseminated POALs. Furthermore, we observed that among our patients with stage IE POALs, lymphoma can be cured after complete resection alone as only 1 (5.5%) of our 18 patients developed local relapse, suggesting that close observation without adjuvant treatment after complete resection is another option for localized POALs.

In terms of the similar EFS and OS, our findings indicated that for patients with localized MALT lymphoma of POALs, the first-line chemotherapy is an effectively alternative treatment for patients who can't receive radiotherapy. In a recent retrospective analysis of 142 patients with stage I MALT lymphoma of POALs, Desai A et al. reported that 22 of 111 patients (108 with CR) receiving first-line radiotherapy alone developed local or systemic relapse, whereas 2 of 7 patients (all with CR) treated with chemotherapy alone had local or systemic relapse [36]. In their study, the prescribed radiotherapy dose ≥ 30.6 Gy was associated with better CR rate and progression-free survival. This is consistent with our study showing radiotherapy alone with a median dose of 40 Gy provided 5-year EFS and OS rates of 89.7% and 90.4%, respectively.

In conclusion, our results demonstrated that systemic chemotherapy alone provided favorable outcomes in patients with localized and disseminated POAL. For patients with localized MALT-POAL, low-dose oral alkylating chemotherapy with or without rituximab is as effective as more intensive regimens. In addition, radiotherapy or surgery can also efficiently control local disease in localized MALT-POAL. Old age and stages III–IV were the most critical prognostic factors for OS. Other cytotoxic chemotherapies such as fludarabine and bendamustine have exhibited efficacy in treating MALT lymphoma [41–43]. Additional studies are required to analyze different combinations of treatment modalities for minimizing long-term toxicity and improving outcomes in patients with poor prognostic factors.

## MATERIALS AND METHODS

### Clinical pathological features and treatment modalities

Patients diagnosed with POALs between January 1990 and December 2015 were retrospectively reviewed. The pretreatment evaluation involved history taking, comprehensive physical examination, complete blood cell count analysis, blood chemistry analysis (including lactate dehydrogenase [LDH]), bone marrow studies, a whole-body CT scan, or MRI for orbital lesions. Since 2006, FDG-PET has been used for aggressive lymphoma staging in our institution. However, the FDG-PET scan in staging of low-grade lymphoma, including MALT lymphoma, is not covered by health insurance. Among 54 patients diagnosed after 2006, 14 patients (26%) underwent FDG-PET for initial staging. The patients with POALs were divided into the following 2 groups on the basis of their disease staging according to the modified Ann Arbor staging [39, 44]. Considering bilateral ocular adnexal lesions of lymphoma and adjacent lymphatics involvement could be treated by local irradiation, the localized POAL (stage IE to IIE1) was defined as a lymphoma involving unilateral ocular adnexal lesions with adjacent lymphatics, or bilateral ocular adnexal lesions with adjacent lymphatics in this study. If primary lesions involved ocular adnexal lesions and non-adjacent lymphatics (i.e. above the diaphragm), we defined them as stage IIE2 POALs. Patients with stage IE–IIE1 disease who received frontline radiotherapy, chemotherapy, rituximab-based immunochemotherapy, or other treatments were included in this retrospective study, whereas those who were treated with first-line antibiotics (including doxycycline) were excluded.

**Figure 5:**
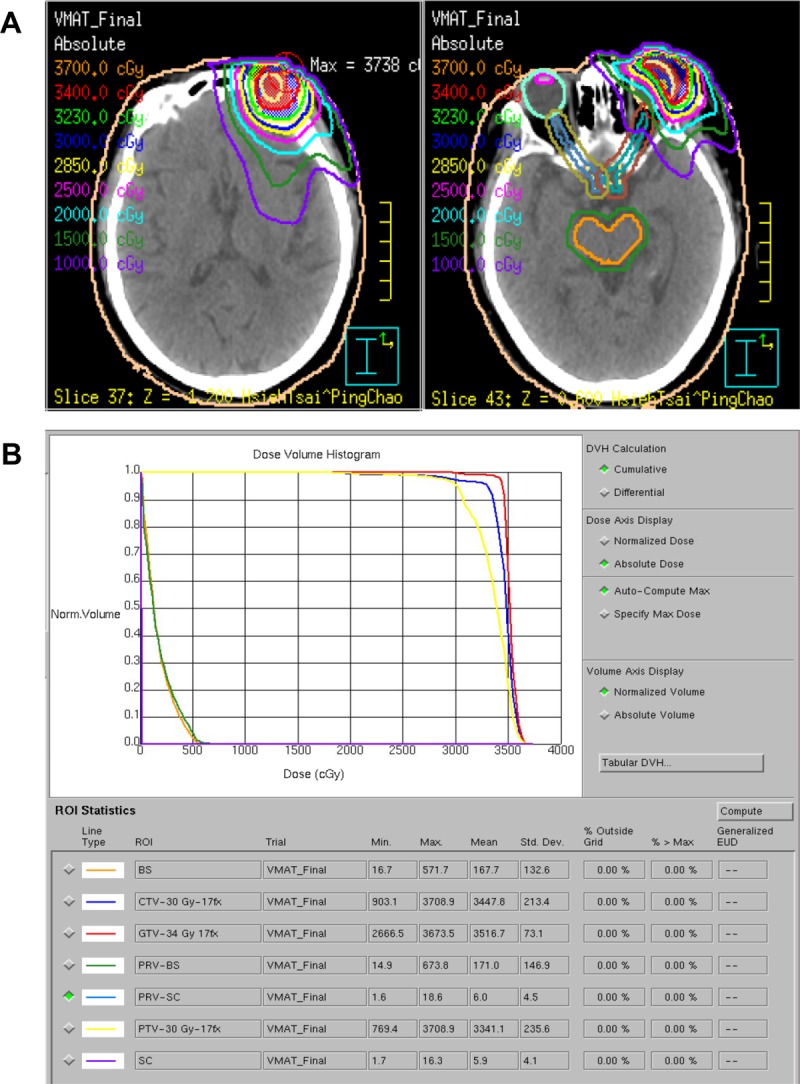
Isodose curves (**A**) and a dose-volume histogram (**B**) of the volumetric-modulated arc therapy plan using coplanar and noncoplanar beam arrangements displayed on the axial plane. The radiation prescription dose was 34 Gy (3400 cGy) in daily fractions of 2.0 Gy for one 79-year-old woman with left orbital MALT lymphoma. Abbreviations: GTV, gross target volume; CTV, clinical target volume; PTV, planning target volume. BS, brain stem; SC, spinal cord.

Among the patients with stage IE–IIE1 disease belonging to the radiotherapy-alone group, most patients were treated with a 6-MV photon beam for POALs of the orbit, extraocular muscle, eyelids, lacrimal gland, or conjunctiva. However, some received a 6–20-MeV electron beam for conjunctival lesions. Of the patients, 30 underwent 2-dimensional radiotherapy; 2 received a 3-dimensional conformal technique; 1 underwent intensity-modulated radiotherapy; and 1 underwent volumetric-modulated arc therapy (Figure 5) (Table 2). The radiation prescription dose ranged from 30 to 50 Gy (median dose: 40 Gy) in daily fractions of 1.8–2.0 Gy.

The patients with stage IE–IIE1 disease in the systemic treatment group were treated with 3 chemotherapy protocols: (1) daily low-dose cyclophosphamide or chlorambucil with or without rituximab (375 mg/m^2^ of rituximab administered every 3–4 wk); (2) cyclophosphamide, doxorubicin, vincristine, and prednisolone (CHOP)-based regimens (the dose of doxorubicin was lowered or omitted on the basis of the attending physicians’ judgment); and (3) R-CHOP-based chemotherapy (CHOP plus 375 mg/m^2^ of rituximab administered every 3–4 wk). Other treatment modalities are listed in Table 2.

Patients with stage IIE2–IVE disease were treated with systemic chemotherapy with or without rituximab or in combination with radiotherapy. The types of chemotherapy regimens and radiation doses were similar to those for the patients with stage IE–IIE1 disease.

### Evaluation and ethic statements

Previous studies evaluating the responses to radiotherapy or chemotherapy for POALs were based on the clinical, radiologic, and pathologic criteria according to the international response criteria for malignant lymphoma [27, 28, 30, 45]. Because POALs are irregularly shaped and the tumors are not centrally located, one ocular lesion (which may involve the eye and both adjacent ocular walls), it is difficult to measure the tumor volume. Some studies suggest that the application of the Response Evaluation Criteria in Solid Tumors (RECIST) and RECIST 1.1 for assessing the treatment response of POALs is valuable [31, 46, 47, 48].

In the present study, the treatment response was assessed based on the primary location of the POALs, which was divided into two subgroups, conjunctival lesions and non-conjunctival lesions. All patients received regular ophthalmologic local examination and a physical examination every 2 to 3 months, and follow-up CT examinations or MRI examinations every 3 months after starting treatment. For conjunctival lesions of POALs, a complete remission (CR) was defined as the complete absence of clinical evidence of lymphoma on slit-lamp examination for at least 6 weeks, whereas a partial response (PR) was defined as a disease reduction of at least 50% for 6 weeks [49]. For non-conjunctival lesions of POALs, the assessment of tumor response was according to the Response Evaluation Criteria in Solid Tumors (RECIST) and RECIST 1.1 [31, 46, 47].

The study protocol was approved by the Research Ethics Committee of National Taiwan University Hospital (NTUH: 201505013RIND). The patients’ medical data were anonymized prior to the access and analysis. The Research Ethics Committee waived the need for written informed consent from the patients because all potential patient-identifying information was removed prior to data analysis.

### Statistical analysis

The discrete variables of the patients with stage IE–IIE1 and IIE2–IVE POALs were compared using the chi-square test or Fisher exact test. If continuous data were not normally distributed, Mann–Whitney *U* tests were used to compare continuous variables and medians of distributions. Event-free survival (EFS) after the first-line treatment was calculated from the date of initial treatment until treatment failure, including disease progression, relapse, or treatment discontinuation for any reason, including death. Overall survival (OS) was calculated from the date of initial treatment to the date of death from any cause [45, 50]. A *P* value of < 0.05 was considered statistically significant.

## SUPPLEMENTARY MATERIALS FIGURE AND TABLES


