# Supplementary material for: Chemotherapy alone is an alternative treatment in treating localized primary ocular adnexal lymphomas
Source: Oncotarget. 2017 Jun 15;8(46):81329–42. doi: 10.18632/oncotarget.18500 (PMC5655287; doi:10.18632/oncotarget.18500)
Supplement: Supplementary file 1 [file oncotarget-08-81329-s001.pdf]

# Chemotherapy alone is an alternative treatment in treating localized primary ocular adnexal lymphomas

## Supplementary Materials

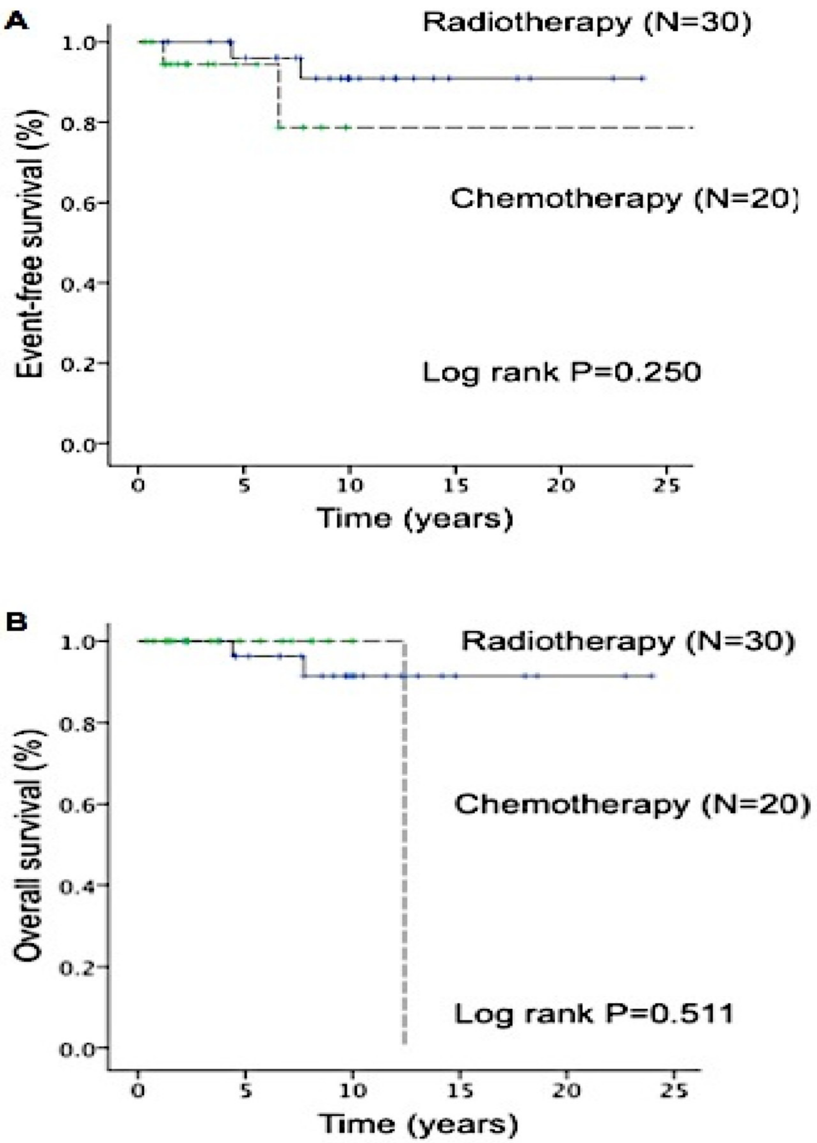


**Supplementary Figure 1: Difference in event-free survival (EFS) and overall survival (OS) between radiotherapy and chemotherapy in patients with stage IE–IIE1 POALs (MALT lymphoma).** (**A**) The 5-year EFS rate did not significantly differ between patients who underwent radiotherapy and those who underwent chemotherapy (96.0% vs 94.4%, $P = 0.250$). (**B**) The 5-year OS rate did not significantly differ between patients who underwent radiotherapy and those who underwent chemotherapy (96.3% vs 100.0%, $P = 0.511$).

**Supplementary Table 1: The complete remission and survival outcome for patients who received different chemotherapy regimens**

| | CR rate | 5-year EFS rate | 5-year OS rate |
|---|---|---|---|
| **Stage I–IIE1** | | | |
| Low-dose alkylating chemotherapy with/without rituximab (*N* = 8) | **87.5%** | **100.0%** | **100.0%** |
| CHOP-based chemotherapy (*N* = 5) | **60.0%** | **100.0%** | **100.0%** |
| R-CHOP–based chemotherapy (*N* = 13) | **76.9%** | **82.5%** | **100.0%** |
| **All stages** | | | |
| Low-dose alkylating chemotherapy with/without rituximab (*N* = 13) | **76.9%** | **60.6%** | **66.7%** |
| CHOP-based chemotherapy (*N* = 7) | **57.1%** | **80.0%** | **100.0%** |
| R-CHOP–based chemotherapy (*N* = 17) | **76.5%** | **87.1%** | **100.0%** |

Abbreviations: CHOP, cyclophosphamide, doxorubicin, vincristine, and prednisolone; R-CHOP, rituximab + CHOP. CR, complete remission; EFS, event-free survival; OS, overall survival.

**Supplementary Table 2: The complete remission and survival outcome for patients with stage IE-IIE1 MALT lymphoma who received different chemotherapy regimens**

| | CR rate | 5-year EFS rate | 5-year OS rate | No. of relapse or progression |
|---|---|---|---|---|
| Radiotherapy (*N* = 30) | 90% | 96.0% | 96.3% | 0 |
| Chemotherapy (*N* = 8) | 62.5% | 100% | 100% | 0 |
| Rituximab-based chemotherapy (*N* = 12) | 83.3% | 91.7% | 100% | 2 |

Abbreviations: CR, complete remission; EFS, event-free survival; OS, overall survival.
